# Suitable transfection methods for single particle tracing in plant suspension cells

**DOI:** 10.1186/1746-4811-10-15

**Published:** 2014-05-31

**Authors:** Janett Göhring, Nick Fulcher, Kurt Schilcher, Andrea Barta, Jaroslaw Jacak

**Affiliations:** 1 Max F. Perutz Laboratories, Medical University of Vienna, Dr. Bohrgasse 9/3, Vienna, Austria; 2 Gregor Mendel Institute, Dr. Bohrgasse 3, Vienna, Austria; 3 Upper Austria University of Applied Sciences, Campus Linz, Linz, 4020, Austria

## Abstract

**Background:**

A multitude of different imaging systems are already available to image genetically altered RNA species; however, only a few of these techniques are actually suitable to visualize endogenous RNA. One possibility is to use fluorescently-labelled and hybridization-sensitive probes. In order to yield more information about the exact localization and movement of a single RNA molecule, it is necessary to image such probes with highly sensitive microscope setups. More challenges arise if such experiments are conducted in plant cells due to their high autofluorescence and demanding transfection procedures.

**Results:**

Here, we report *in planta* imaging of single RNA molecules using fluorescently labeled molecular beacons. We tested three different transfection protocols in order to identify optimal conditions for transfection of fluorescent DNA probes and their subsequent detection at the single molecule level.

**Conclusions:**

We found that an optimized heat shock protocol provided a vastly improved transfection method for small DNA molecules which were used for subsequent single RNA molecule detection in living plant suspension cells.

## Background

Information about the distribution and spatio-temporal dynamics of distinct RNA molecules helps to gain a deeper understanding of a multitude of complex biological processes (e.g., post-transcriptional regulation of gene expression via splicing [1,2], effects of RNA interference [3], or RNA’s complex interaction with chromatin [4]). *In vivo* imaging of RNA has traditionally required usage of overexpressed tagged proteins, saturated probes, or other reporter systems [5-9]. Often, it is challenging to show that these artificially introduced reporters do not have a negative effect on cellular functions, or a biasing effect on raw data. Labelling using hybridization-sensitive probes would, therefore, provide a nearly unperturbed examination of single endogenous mRNA molecules. In many standard biochemical experiments, bulk populations are analyzed instead of populations of single cells. Subtle and often very interesting effects will only be observable in small subpopulations [10]. A prerequisite for such population statistics is, of course, to refrain from following the dogma of the ‘representative cell’ as the outcome of analysis.

There are several advantages that come with studying biological processes at a single molecule level. Single molecule tracing experiments give accurate information about forces that determine the characteristic motion of an individual molecule. Single molecule diffusion parameters calculated within the fragmented subcellular space (e.g. organelles/compartments [11]) can be a powerful tool to decipher cellular processes (e.g. protein interactions in cell signaling [12,13]) or to characterize cell subpopulations [14]. Typically, determination of a single particle trace of an RNA, i.e. one molecule’s diffusion in real time, provides information about direction of movement, step size, and whether there are confined volumes allowing movement within the observed cell volume [15,16]. By analyzing enough molecules, it can also lead to insights about the percentage of mobile fractions and which kind of movement pattern they are following.

Recently, we successfully used fluorescent hybridization-sensitive probes (Molecular Beacons, MBs) to monitor the distribution of mRNA molecules in living plant cells [17]. A simple workflow followed by a robust statistical pipeline led to the establishment of a tool which provides quantitative information on *in vivo* RNA localization and abundance. Due to their special conformation, MBs only fluoresce upon binding to their target sequence. This feature makes MBs the ideal tool for imaging of endogenous RNA in living plant cells as it provides a better signal-to-noise ratio; this is beneficial since autofluorescence in plants is, compared to other organisms, drastically increased [18]. However, a severe disadvantage of MBs is the fact that they need to be delivered into the cell. Hitherto used techniques include microinjection and biolistic bombardment [19,20] which are both laborious, time consuming, and expensive. Moreover, cells become considerably stressed via these invasive transfections. More advanced transfection protocols for mammalian cells make use of nanopore technologies and microfluidics [21,22]. But, nonetheless, microinjection remains the only convenient method for transiently transfecting intact plant tissue.

In order to use MBs for RNA imaging in living plant protoplasts, we needed to establish a gentle transfection protocol. We chose to test polyethylenglycole (PEG), electroporation, and heat shock mediated transfection methods to investigate the localization of a specific MB against the transcripts of RS2Z33, a plant-specific SR protein, which has been described elsewhere [23,24].

## Methods

### MB design

Molecular Beacons are DNA oligonucleotides with a stem loop structure, whose 5’end is conjugated to a fluorophore (Atto550), and its 3’end to a quencher (BHQ2). The loop sequence targets the RNA of interest (RS2Z33 transcripts), whereas the self-complementary stem keeps the quencher and the fluorophore in close proximity, suppressing any signal. If the MB binds to the target, the stem opens and the fluorophore emits a signal. The MB was designed according to Gohring et al. [17]. 33mRNA_EJ6 (5’-Atto550-GATCGCGCGTGATCGGCTGTAGCTTCGGCCGCGATC-BHQ2-3’).

### Cell Culture, Isolation of Protoplasts, and PEG transfection

*Arabidopsis* cell suspension culture protoplasts (derived from 10d old Col-0 seedlings) were prepared and immediately used for PEG transformation as described in Lorkovic et al. [25].

### Electroporation

The optimization procedure included:

– Variation of the electroporation buffer in respect to osmolarity, electrical conductivity, and resistance.

– Variation of the cell density, washing buffer (PIB or GM buffer, for final buffer composition see below), length of incubation on ice after electroporation

– Variation of electrical parameters: voltage, capacity, transferred energy, pulse lengths and intervals.

Further details can be found in the Additional file 1: Data S1. The criteria for the experimental outcome were subjective and included the evaluation of the cellular morphology, presence of cell debris, cell viability, and the state of transfection.

Two hours after protoplast isolation, cells were transfected by electroporation. Approximately 1×10^5^ protoplast cells were washed with 0.275 M Calcium-nitrate and pelleted in a microcentrifuge (150 g for 2 minutes). For electroporation, 0.7 M mannitol (~700 mOsm) was mixed to a final concentration of 500 nM MB. A final volume of 800 μL in a 4 mm cuvette (Biozym Scientific, Oldendorf) was pulsed with following settings of the device Easyject Optima, EquiBio: 240 V, 75 μF, 2 pulses with the length of approximately 7.5 ms and an interval of 20 s. Subsequently, cells were fed with 0.34 M GM-buffer [3163 mg/L Gamborg B5 powder including vitamins (Duchefa), 170 mM D-glucose, 170 mM D-mannitol, 1 mg/L 2.4D, pH 5.5 adjusted with KOH], incubated at room temperature, and kept in the dark for 24 h. Shortly before measurement, cells were washed twice with GM-buffer.

### Heat shock

The optimization procedure included:

– Variation of the osmolarity of the PIB buffer (see below), in order to test the mechanical stress limits of the cellular membrane.

– Variation of the cell density, heat shock temperature, length, and subsequent length of incubation on ice.

– Variation of the washing buffers (PIB or GM buffer, for final buffer composition see below).

Further details can be found in the Supplementary Data S1. The criteria for experimental outcome were subjective and included the evaluation of the cellular morphology, presence of cell debris, cell viability and the state of transfection.

Two hours after protoplast isolation, cells were transfected by heat shock. The protocol was adjusted from Hicks et al. [26]. Approximately 1×10^5^ protoplast cells were resuspended in 200 μL 2×PIB buffer [2 mM MgAc, 50 mM KAc, 5 mM NaAc, 2 mM PMSF, 20 mM HEPES pH7.2, 1 mM DTT, 225 mM mannitol, 125 mM Spermin, 125 mM Spermidin]. Cells were incubated on ice in the dark for 10 minutes and subsequently pelleted in a microcentrifuge (150 g for 2 minutes). The pellet was resuspended in a solution containing 500 nM MBs in 2xPIB buffer and heat shocked for 30 minutes at 28°C in the dark. Afterwards, cells were immediately put on ice for 4 minutes and 100 μL of 1xPIB were added as fast as possible. Eventually, cells were fed with 0.34 M GM-buffer [3163 mg/L Gamborg B5 powder including vitamins (Duchefa), 170 mM D-glucose, 170 mM D-mannitol, 1 mg/L 2.4D, pH 5.5 adjusted with KOH], incubated at room temperature, and kept in the dark for 24 h. Shortly before measurement, cells were washed twice with GM-buffer.

### Imaging of single molecules and analysis

The images were taken on a modified Olympus IX81 inverted microscope. The samples were illuminated through an Olympus UApo N 100×/1.49 NA oil objective with diode laser at 532 nm (Cobolt Calypso 100 TM). The signal acquisition was carried out on an Andor iXonEM + 897 (back illuminated) EMCCD (160 nm pixel size). The experiments were performed using excitation powers of 0.025 kW/cm2 at 532 nm. The samples were illuminated for 5 ms with 35 ms delay time. The illumination protocols were timed with a custom made LabView^®^ based control software. Filter: Overlay (642/532), Dichroic filter: Cy3/Cy5, Emission-filter: Cy3/Cy5 + Bandpass 595/50 (Chroma). The cells were imaged in two illumination configurations, the widefield and highly inclined and laminated optical light sheet (HILO) illumination. The light sheet illumination reduces background fluorescence within the cell, originating from scattered light or other fluorescent molecules [27].

### Analysis

Given the heterogeneous background, we have chosen a detection method based on the isotropic undecimated wavelet transform (IUWT) [28,29]. Wavelet thresholding offers a robust solution for the detection of small bright features, e.g. detection of sub-cellular structures labeled by fluorescent dyes. Since a fluorescent dye can be considered a point light source, its image is the point spread function (PSF) of the optical system that can be approximated by a two-dimensional Gaussian shape.

The typical noise model includes the photon count noise, following a Poisson distribution, and an additive Gaussian read-out noise:

$$n\sim \text{Poi}\left(N\right)*\text{Gauss}\left(0,\sigma \right),$$

With *n* the number of detected photo-electrons and *N* the number of emitted photons.

For an EMCCD camera, the model including the effect of the multiplier can be approximated (as described in [30]) by the formula:

$$p\left(n,N\right)\sim \text{Gauss}\left(\mathrm{GN},\mathrm{FG}\sqrt{\left(N\right)}\right)=\frac{1}{\sqrt{2\pi F^{2}G^{2}N}}e^{\left(\frac{-{\left(n-\mathrm{GN}\right)}^{2}}{2F^{2}G^{2}N}\right)}$$

Where *F* represents the excess noise and *G* the average multiplier gain.

In order to deal with the heterogeneity of the noise, a variance stabilizing transform is applied prior to the IUWT [31]. This step significantly reduces the background and improves the quality of single molecule localization. The method relies on a successive fitting of the noise with a constant kernel wavelet matrix [32]. Subsequently, the fitted background image is subtracted from the original image. After several iterative repeats position of candidates for Gaussian fitting are chosen [28,33,34]. Subsequently, the detected molecule’s position was determined with subpixel accuracy by least square fitting of a 2D Gaussian function in the neighborhood of the detected significant pixels [33-36].

## Results

**Figure 1:**
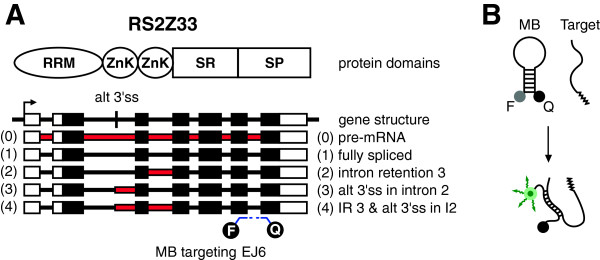
**The scheme depicts the protein and gene structure of RS2Z33 and its transcripts. A)** The Molecular Beacon was designed to bind to exon junction 6 of RS2Z33; i.e. all splice variants of RS2Z33 are targeted. Red – intronic sequences; Black – exonic sequences; white – untranslated regions; RRM – RNA recognition motif; ZnK – Zink knuckle; SR – Serine/Arginine rich domain; SP – Serine/Proline-rich domain; alt 3’-ss – alternative 3’ splice site; IR – intron retention; I2 – intron 2; MB – Molecular Beacon; EJ6 – exon junction 6; F – fluorophore; Q – quencher. **B)** Illustration of the work principle of MB.

The aim of this study is to determine the most suitable transfection method for introducing MBs into living plant suspension cells, preferably reducing the applied stress to the cells. It needs to allow subsequent imaging of MB-labeled RNA at the single molecule level; an optimization for probe concentration and light scattering was, therefore, necessary (strongly depends on debris that attaches to membranes of healthy cells). We decided to test three different transfection approaches: PEG transfection, electroporation and heat shock. In each experiment, wild type *Arabidopsis thaliana* cell culture protoplasts were transfected with fluorescently labeled MBs (Atto550-dye and BHQ2-quencher), which target the exon junction 6 of RS2Z33 (see Figure 1 for a detailed gene structure and the splice variants targeted by the described MB). Exon junction 6 of RS2Z33 is present in all known transcripts of the gene and, thus, can be used as ideal MB target for demonstrating single molecule imaging.

**Figure 2:**
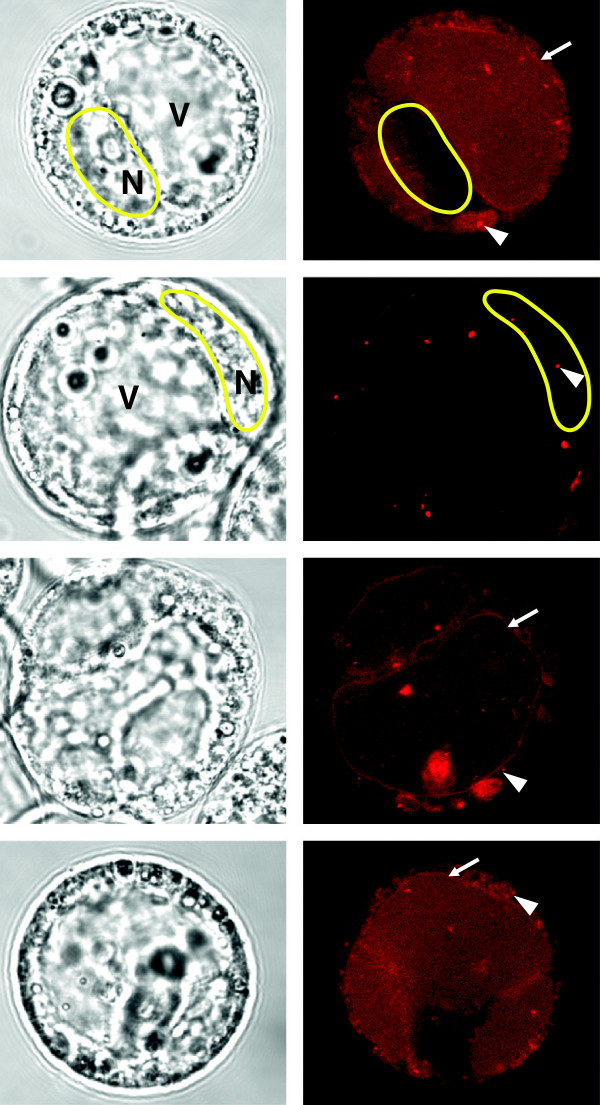
**Transfection of MBs via the standard PEG protocol leads to unspecific conglomerates within the cell.** Representative images of protoplasts transfected with a MB labeled with an Atto550 fluorophore targeting exon junction 6 (Ej6) mRNA of RS2Z33. Arrowheads label foci of PEG-MB conglomerates. Arrows depict vacuolar membranes which are also fluorescently labeled by PEG-MBs. Note the difference between the strongly (1st, 3rd and 4th row) and poorly transfected cells (2nd row). On the left side are the transmission images with visible nucleus (marked yellow). V - vacuole; N - nucleus.

For plant suspension cells, standard transient PEG transfection protocols [37] yield high transfection efficiency and sufficient viability, and compared to the mentioned alternatives have low material demands (e.g., microinjection, bombardment). Figure 2 displays four representative plant protoplasts after transfection of the MB using PEG 6000. Specific signal of the MB was detected on a subcellular level and demonstrates that small oligonucleotides can be introduced into living plant cells using PEG as DNA carrier. The MBs, however, remained unspecifically attached to the PEG and stayed in an open state which introduced strong fluorescence artefacts inside the cell (Figure 2, right panel) as well as fluorescent labelling of the vacuolar membrane in strongly transfected cells (Figure 2, first row). To ensure, that the fluorescent signal originates from the MBs, we measured the autofluorescence of the plant cells which were either transfected only with PEG 6000 (Additional file 2: Figure S1) or with PEG and the MB targeting EJ6 of RS2Z33 (Figure 2 and Additional file 2: Figure S1). None of the cells displayed any specific fluorescence signal arising from PEG itself, but multiple fluorescent artefacts were observed in cells transfected with MBs via the PEG procedure. We were surprised to find that the standard transformation protocol using PEG (polyethylene glycol) is not suited for introducing MBs into living suspension cells. PEG is an unspecifically binding DNA carrier and, unfortunately, triggers the MB to assume open conformation which results in strong fluorescent emission. Another outcome of this experiment is that PEG seems to unevenly distribute throughout the living plant cell, forming fluorescent artefacts and attach to the vacuolar membrane. Since PEG is used as standard transfection substrate, care should be taken depending on the experimental design and question.

**Figure 3:**
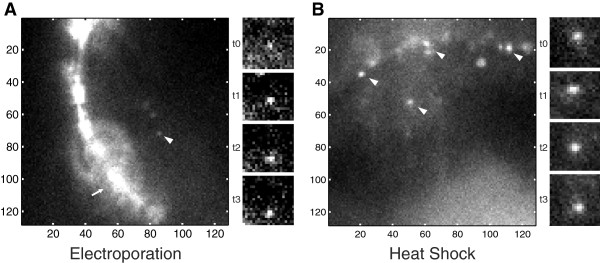
**Electroporation of MBs allows the detection of single molecules and single molecule tracing.** Cells were illuminated via HILO to reduce the background fluorescence. **A)** The overview image shows an electroporated cell. Notice the high fluorescent signal arising from cell debris attached to the cellular membrane. The right panel contains details of a detected single molecule signal in consecutive time frames t0 to t3 (SNR 6,7 ± 2) (5 ms illumination and 30 ms delay between each time frame). **B)** Heat shock transfection of MBs allows the detection of single molecules. The overview image shows a heat shock treated cell. Since the signal-to-noise-ratio of the detected single molecules is 10.1 ± 0.23, the heat shock performed better than electroporation. The right panel contains details of a detected single molecule signal in consecutive time frames t0 to t3 (5 ms illumination and 30 ms delay between each time frame). The arrowheads points to signals of single molecules and the arrow demarks the signal arising from extracellular debris.

**Figure 4:**
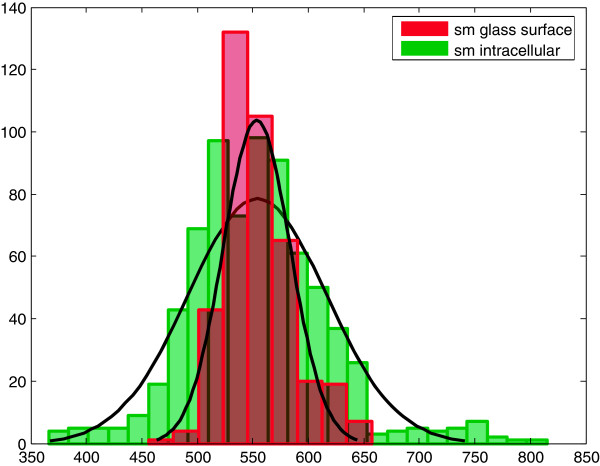
**Distribution of single MB signals.** Statistical distribution of fluorescence intensity counts per fluorescent spot during 5 ms illumination time, obtained from: sparsely and randomly distributed MBs on glass and (red) from single fluorescent spots inside plant cells (green). The single molecule signal distributions have a high similarity, consequently the signal inside the cells originated from fluorescent MBs. Number of cells: 21, Number of signals: 1000. sm - single molecule.

Another way to transform plant protoplasts is by electroporation [38,39]. Interestingly, none of the commercially available electroporation systems are adapted for *A. thaliana* protoplastic cells. Therefore, we needed to optimize the protocol in respect to electroporation buffers, used DNA mass, pulse length, and recovery (for more details and the final protocol see methods). Since MBs are by far smaller than plasmids, which are usually used for transfecting cells, we aimed to increase transfection efficiency for small DNA molecules and additionally to minimize cellular stress. With the optimized parameters, the cells showed good transfection efficiency which allowed confocal imaging [17]. High heterogeneity of the transfection efficiencies was observed; however, we could show that cells with low transfection efficiency are suitable for imaging of single molecules (Figure 3A). The density of molecules is critical for single molecule tracking since higher concentrations impede the identification of the path of one distinct molecule. In order to identify the origin of the signal peaks inside a cell, their average intensities were compared to signals of individual MBs on glass. For the comparison, MBs were immobilized on a glass-substrate. Due to the DNA-glass interaction, a certain percentage of the MB opened and became fluorescent. The intensities of the opened MBs have been compared to the signals obtained inside a cell. The comparison showed a high similarity of the single molecule intensity distributions (Pearson correlation coefficient is 0.3) (Figure 4). Due to the 3D single molecule MB signal distribution inside the cell, the histogram is broadened relative to the signals analyzed on a glass slide. Such intensity histogram broadening can be attributed to the deviation of the single molecule position in the focal plane. Eventually, the MB-transfected cells were measured using a single-molecule sensitive setup with HILO illumination. However, cell debris of dead cells attached to the cell surface, and increased fluorescence and light scattering along the cell membrane led to a strong extracellular background (Figure 3A). This led to a reduced single molecule signal. The high single-molecule brightness and the low background noise within a diffraction limited region obtained within an area next to the single molecule allow for reliable (signal-to-background noise ratio 7 ± 2, SNR averaged over all signals) detection of fluorescent MB molecules. In summary, electroporation enables transfection of MBs allowing subsequent single molecule imaging. However, the heterogeneity in transfection efficiency as well as background and light scattering led to a strong impact on single molecule SNR. Therefore, we decided to search for a gentler transfection method, to reduce extracellular cell debris and to yield just a small amount of labeled mRNAs within the cell (i.e. low transfection efficiency).

The transfection of MBs via heat shock turned out to be a compromise. We optimized the heat shock protocol (oriented along the permeability protocol from Hicks et al. [26]) in respect of used buffers, heat shock, and recovery time, as well as amount of DNA initially used per cell (see Methods for more details). The investigated cells exhibited good viability with relatively low transfection efficiency (with clearly separated single molecule signals). The detected fluorescent signals were at or below the detection limit of a standard laser scanning microscopy setup. We observed relatively high transfection homogeneity within the cell population, good viability, and a low fluorescent background (comparable to non-transfected cells) (Figure 3B). The average SNR of detected MB single molecule signals in cells was 9.7 ± 2.4 (SNR averaged over all signals), which is sufficient for any single molecule fluorescence experiment e.g. single molecule tracking (Figure 3 and Additional file 3: Figure S2). The transfection efficiency shows a clear dependence on concentration and incubation time, which can be used for a relatively precise adjustment of MB concentration. Consequently, this method seems to be a good approach for transfection of cells with MBs permitting single molecule analysis.

## Conclusion

Herein, we report the establishment of two transfection methods for *A. thaliana* suspension protoplasts optimized for measuring Molecular Beacons with single molecule sensitivity. The standard PEG-based transfection method is not applicable for fluorescent microscopy since PEG-bound MBs remain in an open state and lead to a considerable increase in background fluorescence. Electroporated cells also tend to exhibit high background due to the increased amount of extracellular debris. However, transfection via heat shock turned out to be the optimal transfection method for MBs. The transfection has low material requirements and a smaller work load compared to other described methods e.g., microinjection or bombardment. Heat shocked cells are vital and not influenced in their morphology. The extracellular background is reduced and cells exhibit low transfection efficiencies which are best suited for single molecule measurement. Moreover, we report for the first time the successful measurement of single molecules within living plant suspension cells.

## Competing interests

The authors declare no competing interests.

## Authors’ contributions

JG and JJ designed the study, carried out the optimization of the transfection methods and performed single molecule detection, and drafted the manuscript. NF, AB and KS conceived of the study, participated in its coordination, and helped to draft the manuscript. Since KS passed away during the preparation of the manuscript, only the remaining authors read and approved the final manuscript.

## Supplementary Material

Additional file 1: Data S1. Optimization protocols for the electroporation and heat shock. Click here for file

Additional file 2: Figure S1. PEG does not lead to increased fluorescence levels within the measured channel. **A)** The cells transfected only with PEG serve as negative control. **B)** Cells transfected with Atto550-conjugated MBs and PEG. Although a very low sensitivity widefield setup (far below single molecule sensitivity) has been used (Axiovert 200M with AxioCam MRm, standard filter set), fluorescent PEG-MB conglomerates (arrowheads) can easily be observed inside the cell. **C)** A transmission image (TM) of the cells transfected with PEG and MBs. MB – Molecular Beacon against exon junction 6 of RS2Z33; TM – transmission image; PEG – polyethylenglykole. Click here for file

Additional file 3: Figure S2. Movie of a heat shock transfected protoplast. The movie from the fluorescent microscope shows a zoom on the nucleus and surrounding area. Single molecules, appearing as bright dots, corresponding to Atto550-labeled MB in open State bound to the target RNA. The illumination time is 5 ms; time between each frame is 30 ms. Click here for file
