# Supplementary material for: Suitable transfection methods for single particle tracing in plant suspension cells
Source: Plant Methods. 2014 May 31;10:15. doi: 10.1186/1746-4811-10-15 (PMC4076440; doi:10.1186/1746-4811-10-15)
Supplement: Additional file 1: Data S1. — Optimization protocols for the electroporation and heat shock. [file 1746-4811-10-15-S1.pdf]

Electroporation

	**1x**
**EP-buffer 1**	150mM KCl<br>4mM CaCl2<br>10mM Hepes (7.3)<br>225mM Mannitol
**EP-buffer 2**	0.7M Mannitol (pH ca 5.5)
**EP-buffer 3**	EP1/EP2 mixture (1:1)(pH ca 5.5)

Optimization

- transfer approximately 1 x 10^5 cells (in sucrose buffer) to a new 2mL tube
- add 3 to 5 vol of Ca(NO3)^2 buffer
- spin (150g 2min RT) and remove supernatant withoug disturbing the pellet
- estimate volume of pellet and add the same volume of 2x EP-buffer
- add to 780µL using 1x EP **[composition of EP-buffer tested]**
- add DNA-solution in 1x EP **[concentration tested]**
- let stand for 1-3min
- transfer to 4mm cuvette
- electroporation: 240V, 75µF, 2 pulses with 20s interval, approximately 7.5ms long **[EP conditions tested]**
- immediately place on ice for 3 min (optional 10min RT) **[temperature tested]**
- add to 1.5mL GM, sucrose or mannitol buffer **[buffer tested]**
- incubate for 24h at 24°C in the dark (not shaking, tubes should lay on the side) **[incubation time tested]**
- wash 1-2x with GM before final measurement
- [optionally] add Topro-3 on-slide for testing cell vitality

				**Eletroporation Conditions**					**Observation of Transfection and Viability**	**Observations after 1d**
	**Variation**		**Probe**	**V**	**µF**	**ms**	**#**	**IV [s]**		
1				200	75	23	3	10	weak transfection, stressed	
2				200	75	23	3	10	no transfection	
3	EP2; in mannitol after pulse		Cat3/1 (15µL 100mM)	400	5	0	1		no transfection	no transfection, normal morphology
4				200	150	11	3	10	weak transfection	good transfection, stressed (large nucleoli)
5				600	15	1.5	3	10	*dead*	*good transfection, dying*
6				300	450	36.6	1		good transfection, stressed	good transfection, stressed
7		EP3		280	600	11.9	1		no transfection, alive	**weak transfection, alive**
8		EP2		280	600	ca.120	1		good transfection, stressed	
9		EP3		350	600	11.7	1		weak transfection, stressed, lost vacuoles	
10	in sucrose buffer after pulse	EP2	Cat3/1 (5µL 100mM)	350	600	126	1		*dead*	
11		EP3		240	750	14.5	1		no transfection, midly stressed	
12		EP2		240	750	180.2	1		weak transfection, alive	
13		EP3		280	1050	17.6	1		no transfection, alive	
14		EP2		280	1050	216.4	1		weak transfection, alive, strong extracellular signal	

15		EP2		400	75	3.3	1		no transfection, alive	**good transfection, alive**
16		EP2		400	75	5.3, 5.1, 5.1	3	20	weak transfection, stressed, lost vacuoles	good transfection, stressed
17		EP2		240	150	7.7	1		no transfection, alive	**weak transfection, mildy stressed, normal morphology**
18	in GM buffer after pulse	EP2	Cat3/1 (15µL 20mM)	240	150	9.3, 8.8, 8.6	3	20	weak transfection, midly stressed	good transfection, stressed
19		EP2		240	100	8,8	1		no transfection, alive	**good transfection, midly stressed**
20		EP2		240	100	10, 10, 8.6	3	20	no transfection, alive, strong extracellular signal	weak transfection, alive
21		EP3		240	525	11.3, 9.2, 8.6	3	20	*dead*	*dead*
22		EP3		364	75	15.8, 12.5, 12.0	3	20	no transfection, alive	no transfection, alive
23		EP3		240	525	1.5,1.5,1.5	3	20	*dead*	*dead*
24		RT after EP		400	75	2.5, 2.2	2	20	no transfection, alive	*dead*
25		4°C after EP	Cat3/1+Scram (15µL 20mM)	400	75	2.5, 2.2	2	20	no transfection, alive	*dead*
26	EP2; in GM buffer after pulse	RT after EP		240	150	5.2, 4.7	2	20	no transfection, alive	no transfection, alive
27		4°C after EP		240	150	5.2, 4.7	2	20	no transfection, alive	no transfection, stressed
28		RT after EP	Cat3/1 (3µL 100mM)	240	150	5.1	1		no transfection, alive	no transfection, alive
29		4°C after EP		240	150	5.1	1		no transfection, mildy stressed	weak transfection, stressed

Conclusion

4°C stressed the cells substantially, recovery should be performed at RT

240V, 150µF, 2, pulse length approximately 5ms does not disturb the cells and leads to weak transfection

In order to increase the probability of transfection the pulse length was increased by reducing the applied capacity to 75µF yielding a pulse length of approximately 7.5ms.

Final protocol

- transfer approximately 1 x 10^5 cells (in sucrose buffer) to a new 2mL tube
- add 3 to 5 vol of Ca(NO3)^2 buffer
- spin (50g 1min RT) and remove supernatant withoug disturbing the pellet
- estimate volume of pellet and add the same volume of 2x EP-buffer
- add to 780µL using 1x EP
- add DNA-solution [20mM each] in 1x EP
- let stand for 1-3min **(**not longer**)**
- transfer to 4mm cuvette
- electroporation: 240V, 75µF, 2 pulses with 20s interval, approximately 7.5ms long
- immediately place on ice for 3 min **(**optional 10min RT**)**
- add to 1.5mL GM buffer
- incubate for 24h at 24°C in the dark (not shaking, tubes should lay on the side)
- wash 1-2x with GM before final measurement
- [optionally] add Topro-3 on-slide for testing cell vitality

Heat shock

Protocol

- 1x10^{5}µL Zellen (in sucrose buffer) + 200µL 2x PIB
- xx min on ice **[incubation time tested]**
- spin down (150g 2min RT)
- tranfer 5µL to 10µL of the pellet to a new tube
- add Oligo (20mM) in PIB **[buffer concentration tested]**
- heat shock **[heat shock parameters tested]**
- put immediately on ice **[incubation length tested]**
- add 100µL 1x PIB
- wash with GM (1-2x)

Final Protocol

- 1x10^{5}µL Zellen (in sucrose buffer) + 200µL 2x PIB
- 10 on ice
- spin down (150g 2min RT)
- tranfer 5µL to 10µL of the pellet to a new tube
- add Oligo (20mM) in 2x PIB
- heat shock: 30min 28°C in the dark
- put immediately on ice (4min)
- add 100µL 1x PIB
- feed cells with GM buffer (add up to 1.5mL)
- incubate for 24h RT in the dark
- wash with GM (1-2x)

Oligos

3µL 100mM cat3/1 oder cat3/2
3µL 100mM respective scramble
1.7µL 50mM MgCl2
add 20µL buffer (3x PIB) + Wasser

		1x	2x	3x
**PIB**	250mM Mg-Acetate	2mM	80µL	120
	1M K-Acetate	50mM	500µL	750
	1M Na-Acetate	5mM	50µL	75
	200mM PMSF	2mM	100µL	150
	1M HEPES (7.3)	20mM	200µL	300
	1M DTT	1mM	10µL	15
	5x Puffer D	1x	2000µL	2400
	H2O		1760µL	1190

		1x	5x
**Puffer D**	Mannitol powder	225mM	2000mg
	Spermin powder	125mM	135mg
	Spermidin powder	125mM	58mg
	H2O	add 10mL	add 10mL

	Variation		Oligo	Heat Shock	Observation after 24h
1	1st 1xPIB, 15' ice, wash in GM		Cat3/1 + scra	15min 23°C	no transfection
2	1st 1xPIB, 15' ice, wash in 1xPIB		Cat3/1 + scra	15min 23°C	no transfection
3	1st 1xPIB, 30' ice, wash in GM		Cat3/1 + scra	15min 23°C	*dead*
4	1st 1xPIB, 30' ice, wash in 1xPIB		Cat3/1 + scra	15min 23°C	*dead*
5	1st 2xPIB, 15' ice, wash in GM		Cat3/1 + scra	15min 23°C	**weak transfection**
6	1st 2xPIB, 15' ice, wash in 1xPIB		Cat3/1 + scra	15min 23°C	**weak transfection**
7	1st 2xPIB, 30' ice, wash in GM		Cat3/1 + scra	15min 23°C	no transfection
8	1st 2xPIB, 30' ice, wash in 1xPIB		Cat3/1 + scra	15min 23°C	no transfection
9	1st 1xPIB, 10' ice, wash in GM, 4µL of pellet		Cat3/1 + scra	15min 23°C	**weak transfection**
10	1st 1xPIB, 10' ice, wash in 1xPIB, 4µL of pellet		Cat3/1 + scra	15min 23°C	**weak transfection**
11	5µL Pellet; 1st 2xPIB; 10' ice	Oligo in 1x PIB	Cat3/1 + scra	15min 23°C	**weak transfection**
12			Cat3/1 + scra	30min 23°C	no transfection
13			Cat3/1 + scra	15min 28°C	no transfection
14			Cat3/1 + scra	30min 28°C	no transfection
15		Oligo in 2x PIB	Cat3/1 + scra	15min 23°C	no transfection
16			Cat3/1 + scra	30min 23°C	no transfection
17			Cat3/1 + scra	15min 28°C	no transfection
18			Cat3/1 + scra	30min 28°C	no transfection
28	Oligo in 2x PIB		Cat3/1	30min 28°C	**weak transfection**
29			Cat3/1	15min 28°C	no transfection
30			Cat3/1	15min 32°C	no transfection
