# Supplementary figures and images for: Suitable transfection methods for single particle tracing in plant suspension cells
Source: Plant Methods. 2014 May 31;10:15. doi: 10.1186/1746-4811-10-15 (PMC4076440; doi:10.1186/1746-4811-10-15)

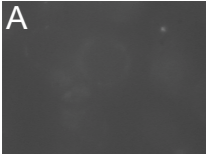

PEG

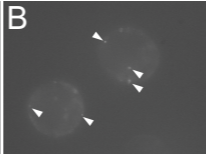

PEG + MB

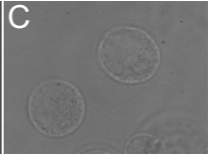

PEG + MB (TM)

Supplement: Additional file 2: Figure S1. — PEG does not lead to increased fluorescence levels within the measured channel. A) The cells transfected only with PEG serve as negative control. B) Cells transfected with Atto550-conjugated MBs and PEG. Although a very low sensitivity widefield setup (far below single molecule sensitivity) has been used (Axiovert 200M with AxioCam MRm, standard filter set), fluorescent PEG-MB conglomerates (arrowheads) can easily be observed inside the cell. C) A transmission image (TM) of the cells transfected with PEG and MBs. MB – Molecular Beacon against exon junction 6 of RS2Z33; TM – transmission image; PEG – polyethylenglykole. [file 1746-4811-10-15-S2.pdf]
